# Carmustine Wafers (Gliadel) in Brain Metastases: Revisiting an Old Concept With New Insights

**DOI:** 10.7759/cureus.91257

**Published:** 2025-08-29

**Authors:** Aparna Suryadevara, Ninh Doan, Samuel Borak, Naresh Bellam, Asad Khan, Vivian Hamlett

**Affiliations:** 1 Internal Medicine Hospitalist, Hospital Physician Services Southeast-PC South, Baptist Medical Center South, Montgomery, USA; 2 Academic Hospitalist, UAB Medicine, Baptist Medical Center South, Montgomery, USA; 3 Radiation Oncology, Gandhi Medical College, Hyderabad, IND; 4 Neurological Surgery, Baptist Health Neuroscience Partners Neurosurgery, Baptist Medical Center South, Montgomery, USA; 5 Pathology, UAB Medicine, Baptist Medical Center South, Montgomery, USA; 6 Hematology/Oncology, Montgomery Cancer Center, Baptist Medical Center South, Montgomery, USA

**Keywords:** brain metastasis, carmustine, gliadel, progression-free survival, survival

## Abstract

Introduction: Brain metastases (mets) are the most common type of brain tumor and a major cause of mortality in cancer patients. Better control of brain mets is therefore crucial for improving both quality of life and survival. Carmustine wafers (BCNU, Gliadel) have been shown to improve local control and survival in patients with high-grade gliomas. In this study, we reviewed patients at our institute who received BCNU wafers for brain mets over the past seven years to assess potential benefits in local disease control and overall survival.

Materials and methods: This was a single-institute retrospective observational study evaluating the use of carmustine (BCNU) wafers following resection of brain mets. Data were retrieved from the pathology department records between 2018 and 2025, including 73 patients. Patients were divided into two groups: Arm A, who underwent brain tumor resection without BCNU implantation, and Arm B, who underwent resection with BCNU wafer placement. Statistical analysis was performed, and a two-tailed p-value < 0.05 was considered statistically significant.

Results: There was an equal proportion of male and female patients, with comparable age distribution in both arms. The most common primary cancer was lung cancer, and the frontal lobe was the most frequent site of mets. Progression-free survival (PFS) and overall survival (OS) were assessed. PFS was significantly higher in patients who received BCNU wafers (p = 0.008), while OS also favored BCNU use but did not reach statistical significance. Survival outcomes were not influenced by the type of primary cancer or the site of brain mets. PFS was significantly better in patients aged ≤ 65 years who underwent resection (p = 0.01).

Discussion: The use of BCNU wafers in brain mets has been reported in only a few studies, mostly with small sample sizes and published before 2015. In our study, we evaluated their role in the modern era of chemotherapy and targeted therapies, many of which have improved penetration across the blood-brain barrier. PFS was 95% at six months, one year, two years, and five years in patients treated with BCNU wafers, compared to 71% at six months and 68% at one and two years in the non-BCNU group. This strongly favors BCNU use after brain met resection. Our study demonstrated higher PFS rates than previously reported, which may reflect the synergistic effect of newer systemic treatment options. While BCNU is expected to improve local disease control rather than OS, we observed an OS advantage in the BCNU group during the first six months, although this did not reach statistical significance. The impact of age on survival was evident, with younger patients faring better, but this effect was independent of BCNU use. Further studies are warranted to explore this parameter in more detail.

Conclusions: Brain mets are most common in lung cancer, and the frontal lobe is the most frequent location. The use of BCNU after resection of brain mets improves PFS across primary cancer types, particularly lung cancer. OS also favored BCNU use, though not significantly. Younger patients (≤65 years) had better survival following surgery for brain mets, with or without BCNU use.

## Introduction

The most common brain tumor is metastasis (mets), occurring in about 25% of cancer patients [1,2]. Symptomatic brain mets develop in 8%-10% of patients [3,4], and this rate is rising with advances in systemic cancer treatment. Although a few newer targeted agents can cross the blood-brain barrier, most systemic therapies do not achieve therapeutic levels in the brain, even when extracranial disease is well controlled. A significant proportion of patients with stage IV cancer die from brain mets. Therefore, better local control of brain mets is critical for improving quality of life and survival, especially in the current era of prolonged survival with advanced systemic therapies [5-7].

Carmustine wafers (BCNU, Gliadel) have been shown to improve local control and survival in high-grade gliomas, but evidence supporting their use in brain mets remains limited [8,9]. Local treatment options for brain mets include surgery, radiation therapy, and intracavitary BCNU placement after resection. The role of repeat radiation therapy is limited by the risk of radiation necrosis, emphasizing the need for alternative local strategies. Recurrence or local failure after surgery alone is often due to microscopic residual disease at the tumor bed or adjacent brain parenchyma. As in malignant gliomas, BCNU wafers may play a role in reducing recurrence and improving outcomes in brain mets [9-11]. Reported local failure rates after surgery alone range from 19% to 46% [12].

The use of BCNU in brain mets has been reported as safe and with some efficacy, but the available literature is sparse and mostly limited to small, single-arm studies conducted before the widespread use of newer systemic therapies.

In this study, we analyzed patients at our institute who underwent BCNU wafer placement following resection of brain mets over the past seven years, to assess their impact on local disease control and overall survival. To our knowledge, this represents the largest single-institution experience with BCNU in brain mets in recent years.

## Materials and methods

This was a single-institute retrospective observational study. Patients who underwent surgery for suspected brain mets at Baptist Medical Center South in Montgomery, Alabama, between January 2018 and January 2025 were identified from pathology records, following approval by the Institutional Review Committee (January 2, 2025). A total of 73 patients were identified. Ten were excluded as final histopathology did not confirm brain mets: five had radiation necrosis/fibrosis, one had an abscess, one had toxoplasmosis, and three had spinal metastases without brain involvement. The final analysis included 63 patients. Data collected included demographics (age, gender), clinical and radiological features, site of brain mets (on CT or MRI), systemic staging (including 18F-FDG PET-CT), primary cancer type, and histopathology of the resected brain lesion. Progression-free survival (PFS) was defined from the date of brain met resection (with or without BCNU wafer placement) until intracranial recurrence or last follow-up. Overall survival (OS) was analyzed both from the time of primary cancer diagnosis and from the time of brain met resection, until death or last follow-up. Patients were divided into two arms: Arm A (n = 28), who underwent resection without BCNU wafer placement, and Arm B (n = 35), who underwent resection with BCNU wafer placement. Group comparisons were performed to assess significant differences between arms. Normality of data was tested using the Kolmogorov-Smirnov test. Appropriate statistical tests were applied to calculate p-values. Survival analysis was performed using the Kaplan-Meier method, with log-rank testing for significance and hazard ratio estimation. All statistical analyses were performed using GraphPad Prism 10.4 (Dotmatics, Boston, MA, USA) and online calculators (socscistatistics; astatsa, Navendu Vasavada, 2016). A two-tailed p < 0.05 was considered statistically significant.

## Results

Patient demographics, along with clinical, radiological, and pathological data, were analyzed in both arms. No statistically significant differences were observed between the groups, as summarized in Table 1. Both arms included an equal proportion of male and female patients. The age at presentation was comparable, ranging from 42-87 years in Arm A and 42-84 years in Arm B.

**Table 1:** Demographic, clinicopathological, and radiological characteristics of patients in the two study arms.

Parameter	Arm A (n = 28)	Arm B (n = 35)	p-value
Gender			0.15
Female	43 %	53%	
Male	57 %	47 %	
Age at presentation	65.6 years (± 11.5)	62.9 years (± 11.5)	
Brain metastasis location			0.61 (other location excluded)
Frontal/frontoparietal	14	20	
Occipital/parieto-occipital	6	9	
Temporal	5	3	
Cerebellar	3	2	
Other location	0	1	
Primary cancer			0.38 (colon and CUP excluded)
Lung	20	20	
Breast	1	6	
Colon	0	3	
Melanoma	3	2	
Renal cell carcinoma	1	2	
Endometrium	2	1	
Carcinoma of unknown primary (CUP)	1	1	
Bladder	0	1	

Primary cancer was recorded based on surgical histopathology (HPE) after brain met resection. Medical records were reviewed to document the initial date of cancer diagnosis, date of brain met diagnosis, date of surgery, date of disease progression in the brain, systemic progression, and date of death. The type and stage of primary cancer at diagnosis, along with the HPE of resected brain mets, were also recorded. Radiological imaging, including 18F-FDG PET, MRI, or CT scans, was reviewed at baseline and during follow-up. Brain imaging at diagnosis, postoperatively, and at all subsequent follow-up or hospitalization visits was assessed until study completion.

The results are summarized in Table 1. Both study arms were comparable, with no significant differences in baseline clinical parameters, allowing for meaningful comparison.

**Figure 1:**
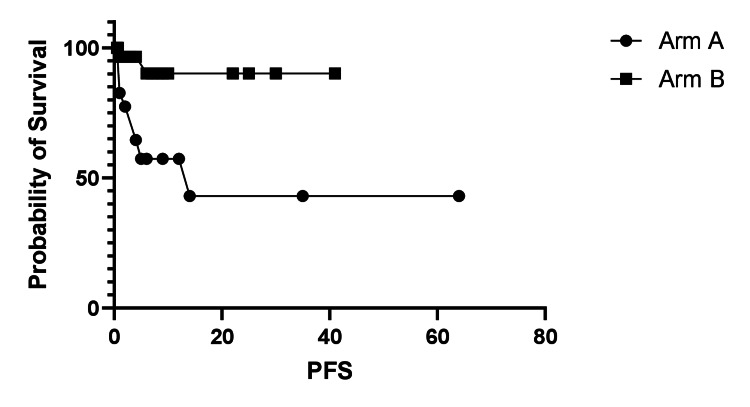
Progression-free survival (PFS) analysis. PFS favored Arm B (BCNU use) with a log-rank (Mantel-Cox) test p = 0.008. Median PFS was 14 months for Arm A and undefined for Arm B. The hazard ratio (Arm A/Arm B) was 5.92 (95% CI 1.79-19.53).

PFS in Arms A and B was compared, as shown in Figure 1, and the analysis favored Arm B.

**Figure 2:**
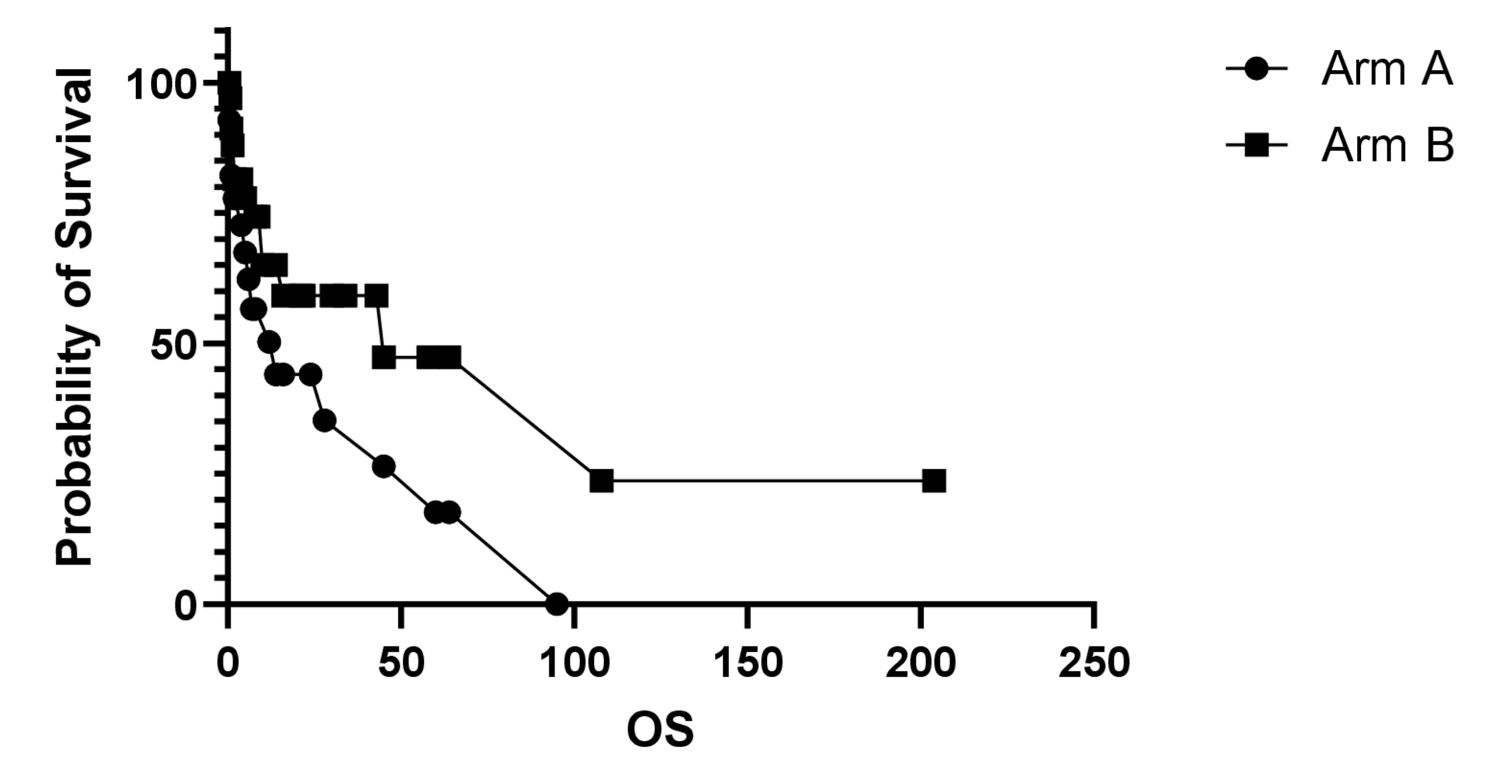
Overall survival (OS). OS was longer in Arm B (BCNU use) compared to Arm A, with a log-rank (Mantel-Cox) test p = 0.06. Median survival was 14 months for Arm A and 45 months for Arm B. The hazard ratio (Arm A/Arm B) was 1.90 (95% CI 0.90-4.02).

OS, measured from the time of cancer diagnosis until death or the end of the study, was compared between the two arms (Arm A and Arm B), as shown in Figure 2. OS analysis favored the BCNU arm.

**Figure 3:**
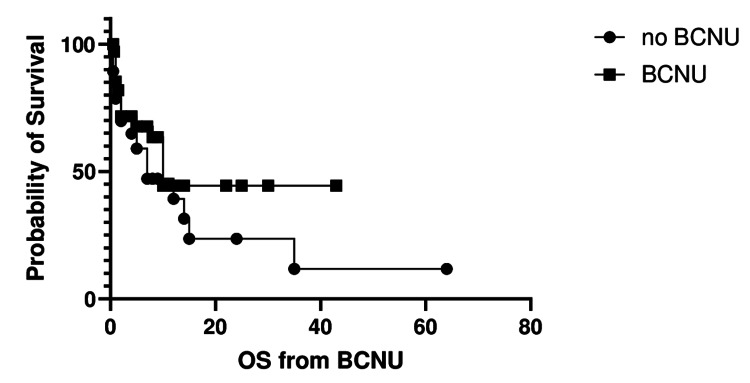
Overall survival (OS) from surgery with or without BCNU use. The OS was analyzed using the log-rank (Mantel-Cox) test, with a p-value of 0.22. Median survival was 7 months for Arm A and 10 months for Arm B. The hazard ratio (Arm A vs. Arm B) was 1.52 (95% CI: 0.74-3.14).

Results for OS from the time of surgery with or without BCNU use were further analyzed. Survival outcomes numerically favored the BCNU arm but did not reach statistical significance, as shown in Figure 3.

We also analyzed survival between the two study arms based on the primary cancer type and the site of brain mets at presentation; however, no statistically significant differences were observed.

**Figure 4:**
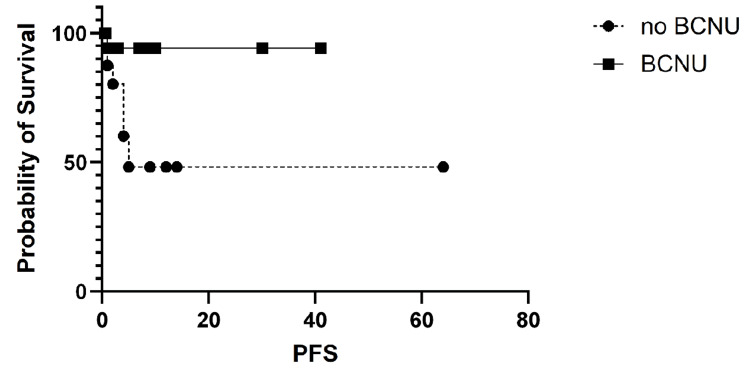
Progression-free survival (PFS) in lung cancer patients. The PFS for patients with lung cancer showed a statistically significant difference, with log-rank (Mantel-Cox) test p = 0.02. Median survival was five months for Arm A (without BCNU) and undefined for Arm B (with BCNU). The hazard ratio (log-rank) for Arm A vs. Arm B was 7.22 (95% CI 1.63-31.9).

PFS was further analyzed by primary cancer type and was not statistically significant overall. The most common primary cancer was lung cancer, and survival in this subgroup was analyzed separately. In patients with lung cancer, the PFS was statistically significant, favoring BCNU use, as shown in Figure 4.

**Figure 5:**
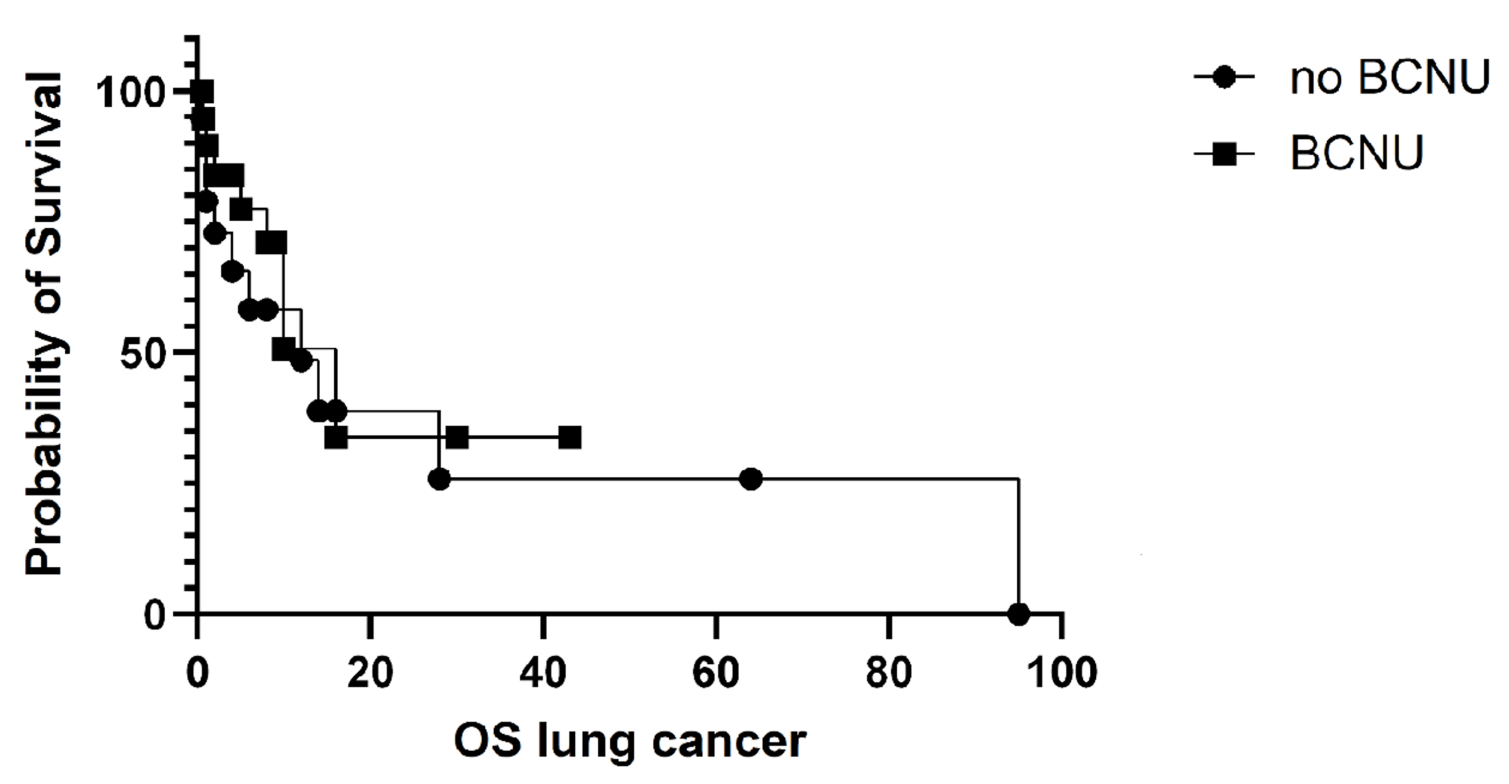
Overall survival (OS) in lung cancer with brain metastases. The OS for lung cancer patients with brain metastases was not significantly different between the two study arms (p = 0.58, log-rank (Mantel-Cox) test). The median survival was 12 months in Arm A and 16 months in Arm B, with a hazard ratio (log-rank; A/B) of 1.26 (95% CI 0.51-3.1).

The OS for lung cancer patients favored BCNU use but was not statistically significant, as shown in Figure 5. 

**Figure 6:**
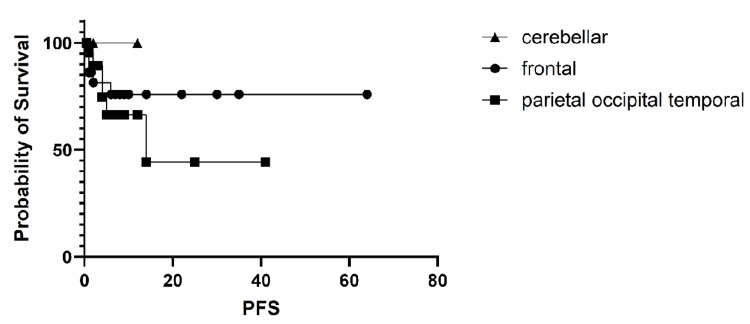
Progression-free survival (PFS) by site of brain metastasis. The variation in PFS according to the site of brain metastases was not statistically significant (p = 0.66).

The PFS and OS in the study arms were analyzed according to the site of brain mets and showed no significant difference. The PFS had a p-value of 0.66, and the OS had a p-value of 0.29. The results for PFS are shown in Figure 6.

**Figure 7:**
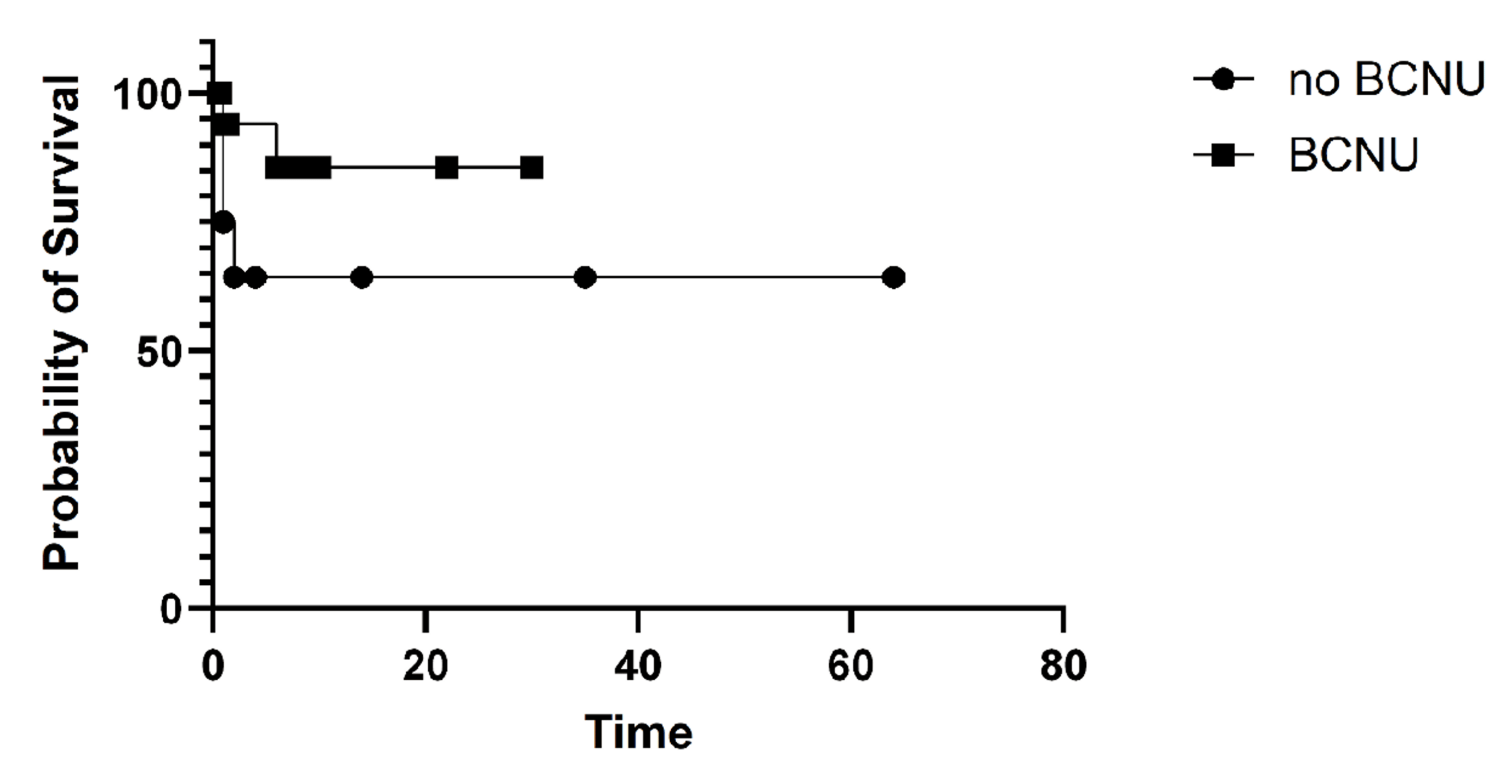
Progression-free survival (PFS) between the two arms in patients with frontal metastases. The PFS was not significantly different between the two arms, with a p-value of 0.12 and a hazard ratio (log-rank; A/B) of 3.31 (95% CI 0.63-17.28).

As most patients in the study had frontal mets, these were further analyzed for PFS and OS, but no significant differences were found. The results for PFS in patients with frontal mets are shown in Figure 7.

**Figure 8:**
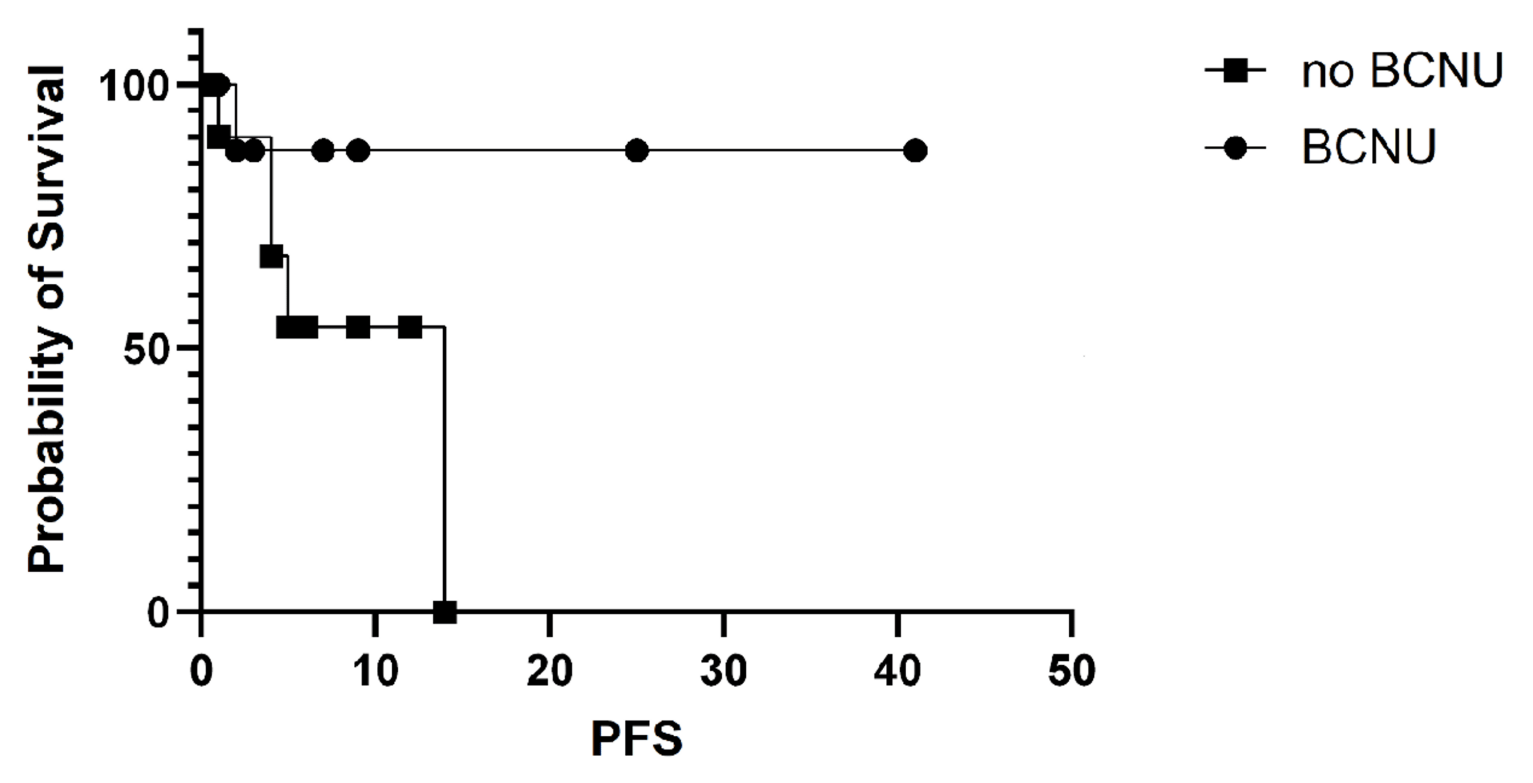
Progression-free survival (PFS) for parieto-occipital metastases between the two arms. The PFS was not significantly different between the two arms, with a p-value of 0.11 and a hazard ratio (log-rank; A/B) of 4.44 (95% CI 0.89-22.08).

The results for PFS in patients with parieto-occipital mets were also not significantly different, as shown in Figure 8.

We analyzed the survival of patients after brain met surgery, with and without BCNU use, in relation to age, as previous literature has reported variable outcomes based on patient age. A statistically significant difference was observed between geriatric patients (>65 years) and non-geriatric patients (≤65 years), with younger patients showing better PFS (p = 0.01). However, in the BCNU arm, there was no significant difference by age for either PFS (p = 0.9) or OS (p = 0.06).

## Discussion

Brain mets occur in about 25%-30% of patients with cancer, regardless of the primary cancer type, according to reports from the United States [1,5]. Of these, around 10% develop symptoms, which impact both survival and quality of life, underscoring the importance of local therapies for brain mets [2-4].

The local recurrence rate after resection alone remains as high as 19%-46%, even with newer chemotherapy and targeted therapies [12-14]. Because of this high rate, local radiation therapy has been recommended based on current evidence [1]. The use of BCNU has been reported only in a few studies with small sample sizes, most conducted before 2015 and prior to the widespread use of newer treatment modalities and chemotherapy [5,8,9]. In our study, we evaluated the benefit of BCNU in brain mets in the context of newer chemotherapy and targeted therapies.

The PFS in our study was 95% at six months, one year, two years, and five years of follow-up, with no local recurrence or progression after six months in the surgical arm with BCNU use. In the arm without BCNU, the PFS was 71% at six months and 68% at both one- and two-year follow-ups. This finding favors BCNU use after brain tumor resection in brain mets. Previous literature has shown variable results with BCNU, ranging from 50% to 100%. For example, PFS was 100% (0 of 25 patients) in a study conducted from 1998 to 2000 by Ewend et al.; 50% (7 of 14 patients) in a study by Ene et al. from 2000 to 2015; and 13% and 30% at 6 and 12 months, respectively, among 31 patients in a study by Mu et al. conducted from 2002 to 2013 [5,8,9]. All of these were single-arm studies without a control group for comparison. In contrast, our study was a two-arm comparison that demonstrates the benefit of BCNU after surgical resection, as it prolongs PFS compared to surgery alone. Our study also reported higher PFS than previously published, which may be attributable to newer systemic treatment options for cancer, as further discussed with OS. In the current study, PFS was compared with the three similar studies available in the literature, as summarized in Table 2.

**Table 2:** Progression-free survival (PFS) in the current study compared with published literature.

Study	Study period	Sample size	PFS at six months	PFS at one year	PFS at two years	PFS at five years
Current study with BCNU use	2018-2025	35	95%	95%	95%	95%
Current study without BCNU use	2018-2025	28	71%	68%	68%	N/A
Ewend et al. [5]	1998-2000	25	100%	100%	100%	N/A
Ene et al. [8]	2000-2015	14	50% (in 0.52-4.29 years)			
Mu et al. [9]	2002-2013	31	87%	70%		

An OS benefit is not expected with BCNU, as it is primarily used for local control of brain mets. Similar findings were observed in our study; however, OS was slightly better in the BCNU arm during the first six months, though the difference was not statistically significant. The median OS from cancer diagnosis was 31 months longer (45 vs. 14 months), and the median OS from surgery with BCNU was 3 months longer (10 vs. 7 months) compared to patients without BCNU. In the study by Ewend et al., the median survival was about 8.1 months (33 weeks), with one-year and two-year survival rates of 33% and 25%, respectively [5]. In the study by Mu et al., OS was 63% at 6 months and 36% at 12 months [9]. In our study, survival at six months, one year, and two years was slightly better in the BCNU arm during the first six months, then became comparable between the two groups. The OS in the BCNU arm was 75% at six months, 64% at one year, and 64% at both two years and five years. In the surgery-only arm (without BCNU), OS was 71% at six months, 68% at one year, and 64% at both two years and five years. The OS findings in the current study are compared with the three similar studies available in the literature, as shown in Table 3.

**Table 3:** Overall survival (OS) in the current study compared with published literature. Showing the OS in current study and literature.

Study	Study period	Sample size	OS at six months	OS at one year	OS at two years	OS at five years
Current study with BCNU use	2018-2025	35	75%	64%	64%	64%
Current study without BCNU use	2018-2025	28	71%	68%	64%	64%
Ewend et al. [5]	1998-2000	25	N/A	33%	25%	N/A
Ene et al. [8]	2000-2015	14	0% (in 1.17-4.71 years)			
Mu et al. [9]	2002-2013	31	63%	36%	N/A	N/A

Overall, survival in our study was higher than that reported in the literature, which may be partly due to the use of newer treatment modalities, as previous studies included patients treated between 1998 and 2015. BCNU treatment may also have contributed to these results, but this requires further evaluation in future studies on brain mets.

The distribution of male and female patients was nearly equal, with a slightly higher proportion of males (60%), similar to the study by Ewend et al. [5]. This likely reflects the predominance of lung cancer as the primary site. In contrast, the study by Ene et al. [8] reported 60% females, as their population had more female-predominant malignancies. In our study, BCNU use did not show a difference in PFS or OS with age, although younger patients (≤65 years) had better OS. While age has been shown to affect survival in recurrent glioblastoma, its impact in brain mets treated with BCNU is contradictory and warrants further investigation, as it could influence patient selection for surgery with BCNU [15,16].

The median age at presentation in our study was approximately 64 years, slightly older than reported in the literature (~52 years) [5,8]. Only one study to date has analyzed survival in relation to age. In that study, PFS, but not OS, favored younger patients with brain mets treated with BCNU [8]. In our analysis, comparing patients aged ≤65 years and >65 years, there was no statistically significant difference in PFS or OS.

The merits of our study include being a single-institute study with all patients treated according to current guidelines, ensuring minimal variation in surgery or cancer treatment. It is also the largest study on brain mets treated with BCNU and, to our knowledge, the only two-arm study in the literature.

The limitations of the study include variability in the primary cancer site, which is inherent in studies on brain mets, although we partially addressed this by analyzing the subgroup of lung cancer patients. Additionally, some patients had very short survival (less than one month), which is expected with brain mets and makes analysis more challenging.

## Conclusions

Brain mets are most commonly seen in lung cancer, with the frontal lobes being the most frequent site. The use of BCNU after resection of brain mets increases PFS across all primary cancers, particularly in lung cancer. OS also trends in favor of BCNU use. Younger patients (<65 years) have better survival when treated for brain mets with surgery, with or without BCNU.

## References

[1] (2025). NCCN Clinical Practice Guidelines in Oncology (NCCN Guidelines®). Central Nervous System Cancers. https://www.nccn.org/guidelines/guidelines-detail?category=1&id=1425.

[2] Fox BD, Cheung VJ, Patel AJ, Suki D, Rao G (2011). Epidemiology of metastatic brain tumors. Neurosurg Clin N Am.

[3] Barnholtz-Sloan JS, Sloan AE, Davis FG, Vigneau FD, Lai P, Sawaya RE (2004). Incidence proportions of brain metastases in patients diagnosed (1973 to 2001) in the Metropolitan Detroit Cancer Surveillance System. J Clin Oncol.

[4] Schouten LJ, Rutten J, Huveneers HA, Twijnstra A (2002). Incidence of brain metastases in a cohort of patients with carcinoma of the breast, colon, kidney, and lung and melanoma. Cancer.

[5] Ewend MG, Brem S, Gilbert M (2007). Treatment of single brain metastasis with resection, intracavity carmustine polymer wafers, and radiation therapy is safe and provides excellent local control. Clin Cancer Res.

[6] O'Day SJ, Gammon G, Boasberg PD (1999). Advantages of concurrent biochemotherapy modified by decrescendo interleukin-2, granulocyte colony-stimulating factor, and tamoxifen for patients with metastatic melanoma. J Clin Oncol.

[7] Carey LA, Ewend MG, Metzger R (2004). Central nervous system metastases in women after multimodality therapy for high risk breast cancer. Breast Cancer Res Treat.

[8] Ene CI, Nerva JD, Morton RP, Barkley AS, Barber JK, Ko AL, Silbergeld DL (2016). Safety and efficacy of carmustine (BCNU) wafers for metastatic brain tumors. Surg Neurol Int.

[9] Mu F, Lucas JT Jr, Watts JM (2015). Tumor resection with carmustine wafer placement as salvage therapy after local failure of radiosurgery for brain metastasis. J Clin Neurosci.

[10] Westphal M, Hilt DC, Bortey E (2003). A phase 3 trial of local chemotherapy with biodegradable carmustine (BCNU) wafers (Gliadel wafers) in patients with primary malignant glioma. Neuro Oncol.

[11] Reithmeier T, Graf E, Piroth T, Trippel M, Pinsker MO, Nikkhah G (2010). BCNU for recurrent glioblastoma multiforme: efficacy, toxicity and prognostic factors. BMC Cancer.

[12] Patchell RA, Tibbs PA, Regine WF (1998). Postoperative radiotherapy in the treatment of single metastases to the brain: a randomized trial. JAMA.

[13] Mahajan A, Ahmed S, McAleer MF (2017). Post-operative stereotactic radiosurgery versus observation for completely resected brain metastases: a single-centre, randomised, controlled, phase 3 trial. Lancet Oncol.

[14] Churilla TM, Chowdhury IH, Handorf E (2019). Comparison of local control of brain metastases with stereotactic radiosurgery vs surgical resection. A secondary analysis of a randomized clinical trial. JAMA Oncol.

[15] Chamberlain MC (2011). Bevacizumab for the treatment of recurrent glioblastoma. Clin Med Insights Oncol.

[16] Nghiemphu PL, Liu W, Lee Y (2009). Bevacizumab and chemotherapy for recurrent glioblastoma. A single-institution experience. Neurology.

